# A learning-based method for drug-target interaction prediction based on feature representation learning and deep neural network

**DOI:** 10.1186/s12859-020-03677-1

**Published:** 2020-09-17

**Authors:** Jiajie Peng, Jingyi Li, Xuequn Shang

**Affiliations:** 1 The School of Computer Science, Northwestern Polytechnical University, Xian, 710072 China; 2 The Key Laboratory of Big Data Storage an Management, Northwestern Polytechnical Universitythe, Ministry of Industry and Information Technology, Xian, 710072 China

**Keywords:** DTIs prediction, Convolutional neural network, Feature representation learning

## Abstract

**Background:**

Drug-target interaction prediction is of great significance for narrowing down the scope of candidate medications, and thus is a vital step in drug discovery. Because of the particularity of biochemical experiments, the development of new drugs is not only costly, but also time-consuming. Therefore, the computational prediction of drug target interactions has become an essential way in the process of drug discovery, aiming to greatly reducing the experimental cost and time.

**Results:**

We propose a learning-based method based on feature representation learning and deep neural network named DTI-CNN to predict the drug-target interactions. We first extract the relevant features of drugs and proteins from heterogeneous networks by using the Jaccard similarity coefficient and restart random walk model. Then, we adopt a denoising autoencoder model to reduce the dimension and identify the essential features. Third, based on the features obtained from last step, we constructed a convolutional neural network model to predict the interaction between drugs and proteins. The evaluation results show that the average AUROC score and AUPR score of DTI-CNN were 0.9416 and 0.9499, which obtains better performance than the other three existing state-of-the-art methods.

**Conclusions:**

All the experimental results show that the performance of DTI-CNN is better than that of the three existing methods and the proposed method is appropriately designed.

## Background

Drug targets are special molecules that can bind to drugs and produce effects in cells, the main molecular targets for drugs are proteins [1]. Drug-target interactions (DTIs) prediction is of great significance for drug repositioning [2], drug discovery [3], side-effect prediction [4] and drug resistance [5]. However, identifying the drug-target interactions via biochemical and chemical biological experiments is costly and time-consuming [6]. Recently, as genomic, chemical, and pharmacological data become more and more complete, new opportunities for identifying drug target interactions have been emerged [2]. Therefore, many researchers have attempted to predict DTIs by using silico or computational approaches to guide in vivo validation in recent years, and thus significantly reduce the cost and time for identifying the drug-target interactions [2].

The traditional computational DTIs prediction approaches are mainly categorized into docking-based approaches [7] [8] and ligand-based approaches [9]. However, the docking is difficult to play a good performance when the three-dimensional structures of the target protein are unknown [10]. The ligand-based approaches are very effective in DTIs prediction, but it often requires a large number of known binding data and thus the prediction results are poor with only a small amount of known data [11].

In recent years, network-based approaches have demonstrated great advantages compared to docking-based and ligand-based methods [12] [13]. First, network-based approaches have a good prediction performance even without the three-dimensional structure of the target. Secondly, they are simple and fast for only by performing simple physical processes [13]. In the past decade, DTIs prediction approaches based on machine learning have also been widely studied. A key idea of these approaches is the assumption that similar drugs may share similar targets [14].Thus predicting DTIs is often regarded as a binary classification issue by using chemical structures of drugs and targets as input features and considering known DTIs as labels [2]. However, most existing prediction methods are limited to homogeneous networks, which ignore a rich variety of topological information and the complex interaction relationship of heterogeneous data [3].

In recent years, a variety of computing methods based on heterogeneous data sources have been developed to predict DTI. *Wang et.al* used a heterogeneous network data to obtain the diffusion feature and directly use the obtained diffusion distributions to derive the prediction scores of DTIs [3]. However, the direct use of diffusion state as a feature or prediction score is vulnerable to the deviation caused by noise and high dimension of heterogeneous network data, resulting in inaccurate DTI prediction [2]. *Luo et.al* provided the DTINet as a new prediction method, which extracted the low-dimensional characteristic information from heterogeneous data sources and used the inductive matrix completion (IMC) approach to predict the drug-target interaction fraction [2]. The inductive matrix completion approach predicts the new DTI by using a small amount of known drug-protein interaction information, combined with the extracted drug and protein characteristic information. This method has been proved to be superior to the commonly used Laplacian regularization least square approach [15], heterogeneous network model [16], cooperation matrix factorization (CMF) [17] and Bipartite local model by learning from local information and neighbors [18].

In this paper, we improve the prediction method by learning low-dimensional vector representations of features from heterogeneous networks, and adopting convolution neural networks (CNN) as classification model. An efficient DTI prediction method is presented, DTI-CNN, which can be used to identify the drug-target interactions, guide biochemical experiments and reduce the cost of research. Here are four major contributions:

- We propose a learning-based method for drug-target interaction prediction that contains three components, named as heterogeneous-network-based feature extractor, denoising-autoencoder-based feature selector and CNN-based interaction predictor.
- Based on random walk with restart (RWR) and denoising autoencoder (DAE) model, DTI-CNN can cope with the noisy, incomplete and high-dimensional features from heterogeneous data sources, including drug, proteins, side-effects and diseases information.
- Based on a deep CNN model, DTI-CNN can handle the low dimensional feature vectors and predict the probability of interaction between each pair of drugs and proteins.
- Based on our DTI prediction task, the results indicate that DTI-CNN is better than the other three state-of-the-art methods and is appropriately designed.

## Methods

**Fig. 1:**
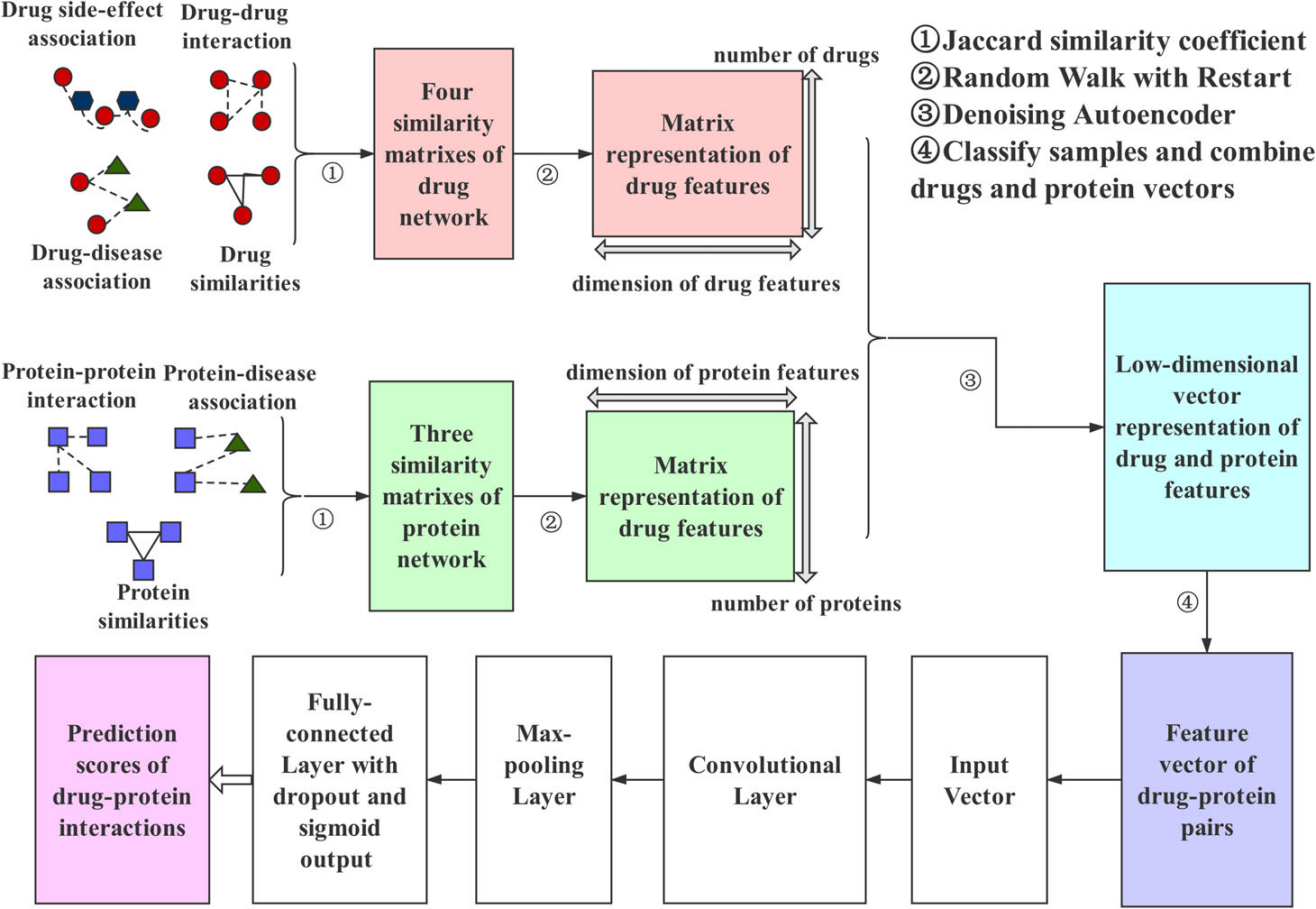
The flowchart of DTI-CNN pipeline. The DTI-CNN contains heterogeneous-network-based feature extractor, denoising-autoencoder-based feature selector and CNN-based interaction predictor. First, the features are extracted from seven networks of drug and protein by the Jaccard similarity coefficient and RWR algorithm, then we get the low-dimensional representation of drug and protein features by adopting the DAE model. Third, a deep CNN model is constructed to predict the interaction of each pair of drugs and proteins

We propose an learning-based method called DTI-CNN to predict drug-target interactions. The workflow of DTI-CNN is shown in Fig. 1. First, the heterogeneous network was constructed by integrating a variety of drug and protein related information sources, and the initial drug feature vector and protein feature vector were obtained by RWR model. In the second step, the high-dimensional features of drugs and proteins are reduced by adopting the DAE model, and the low-dimensional representations of them are obtained respectively. Finally, according to the known drug-protein interactions, the samples are divided into positive samples and negative samples. Combining the feature vector of drug-protein pairs, CNN was adopted to predict the association between each pair of drugs and proteins.

### Heterogeneous-network-based feature extractor

The heterogeneous networks is constructed based on two types of networks as follows. One is the drug-related networks, including drug-drug interactions, drug-disease associations, drug-side-effect associations, drug similarities (based on chemical structures of drugs) [2]. The other is protein-related networks, including protein-disease associations, protein-protein interactions, protein similarities (based on primary sequences of proteins) [2]. Firstly, the Jaccard similarity algorithm [19] is executed on each association and interaction matrix respectively, so we can generate a similarity matrix for each network.

Taking the drug-disease interactions matrix as an example, two sets *A* and *B* are given as two rows in the adjacent matrix, which represent the interactions between two different drugs and all diseases. The Jaccard similarity coefficient[20] is an indicator of the similarity between two sets, defined as follows:

1 $$ Sim(A,B) = \frac{{\vert}A{\cap}B{\vert}}{{\vert}A{\cup}B{\vert}}.  $$

The original adjacent matrix is a description of the relationship between a single row and column node, and the Jaccard similarity coefficient calculation is based on the adjacent two row vectors of the original adjacent matrix. Thus the similarity matrices *S* represent the similarity between each drug or protein node and all features of the column nodes. The element *S*_*i*,*j*_ represents similarity of row *i* and row *j* in the original adjacenct matrix.

After all the original data is transformed into similarity matrices, the RWR algorithm [21] is applied to each similarity matrix, which represents a weighted network. The diffusion state of each drug or protein is obtained on each network, which includes the topological structure relation of each drug or protein with all the other nodes in the network.

The reason for using RWR is that the similarity matrix obtained in the previous step only calculates the similarity of the two nodes in isolation. RWR can be used to consider global structure information in the network. If the distribution state of the two nodes is close, they can be considered to be in a similar position relative to other nodes in the network. According to the RWR principle, the greater the similarity between the two nodes, the higher the transition probability of them [22].

Taking the drug-disease similarity matrix *A*_*i*,*j*_ as an example, we can get the drug-disease transition probability matrix *B* according to the *A*_*i*,*j*_, whose elements *B*_*i*,*j*_ describe the transition probability from the drug node *i* to the disease node *j* [23], that is defined as follows:

2 $$ B_{i,j} = \frac{A_{i,j}}{\sum_{j}A_{i,j}}.  $$

Next, the final drug-disease diffusion state matrix can be obtained by iterative convergence as follows:

3 $$ S_{i}^{t+1} = (1-p_{r})S_{i}^{t}B+p_{r}e_{i}.  $$

Where $S_{i}^{t}$ is the result after *t* iterations, and each element stores the probability of accessing a disease node from the drug node *i* after iteration in the process of random walk, *p*_*r*_ is the restart probability, and *e*_*i*_ is an n-dimensional unit matrix.

After all the similarity matrices is transformed into diffusion state matrices, we splice the single diffusion state matrix of drug and protein networks, so that we can get two diffusion state matrices about drug and protein. The row of the drug diffusion matrix represents different drugs, and the column represents the four nodes of drugs, diseases, side effects and drugs, in which the element *D*_(_*i*,*j*) represents the transition probability between drug *i* and node *j*. The row of the protein diffusion state matrix represents different proteins, and the column represents the three nodes of protein, disease and protein, in which the element *P*_(_*i*,*j*) represents the transfer probability of protein *i* and node *j*.

### Denoising-autoencoder-based feature selector

**Fig. 2:**
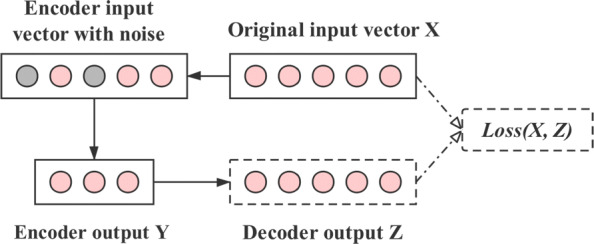
The schematic illustration of denoising autoencoder. The original input data is high-dimensional, noisy and incomplete, the DAE adds noise to it and makes the self-encoder learn to remove the noise, which makes the encoder learn more robust and low-dimensional representation in the input data. Then the decoder is used to recover the original input from low-dimensional data, the loss between the original input and the decoder output is optimized by the RMSProp algorithm

The vector of diffusion state matrix obtained in the previous step is high-dimensional, noisy and incomplete. In order to obtain the essential features, we apply a DAE model which carry on the data operation on the basis of the autoencoder. The main idea of DAE is shown in Fig. 2. Taking the diffusion state matrix about drug as an input example, by adding noise to the input training data and making the self-encoder learn to remove this noise, the real input which has not been polluted by noise can be obtained [24]. Therefore, the encoder can obtain the most essential features from the original input to get more robust representation. This is why its generalization ability is better than that of the general encoder [25]. Autoencoders use automatic Encoders to obtain low-dimensional data through neural networks based on the input data. Similarly, the Decoders to recover the original input from low-dimensional data [26].

In the model, we reduce the dimension of drug features to 100 dimensions and protein features to 400 dimensions. We set the noise figure to 0.2, and use the softplus [27] and RMSProp fuction [28] to optimize the mean-square error (MSE) [29]. At last, the backpropagation (BP) algorithm is used to train our DAE [30].

### CNN-based interaction predictor

Convolutional Neural Networks is a classical and widely used structure since 1980s [31] and can greatly reduces the complexity of convolution neural network [32]. The parameters of each layer network are shared and the number of parameters to be trained is reduced during training. Compared with the standard fully connected neural network, it has better performance in image classification, sentence classification and other classification tasks [33].

**Fig. 3:**
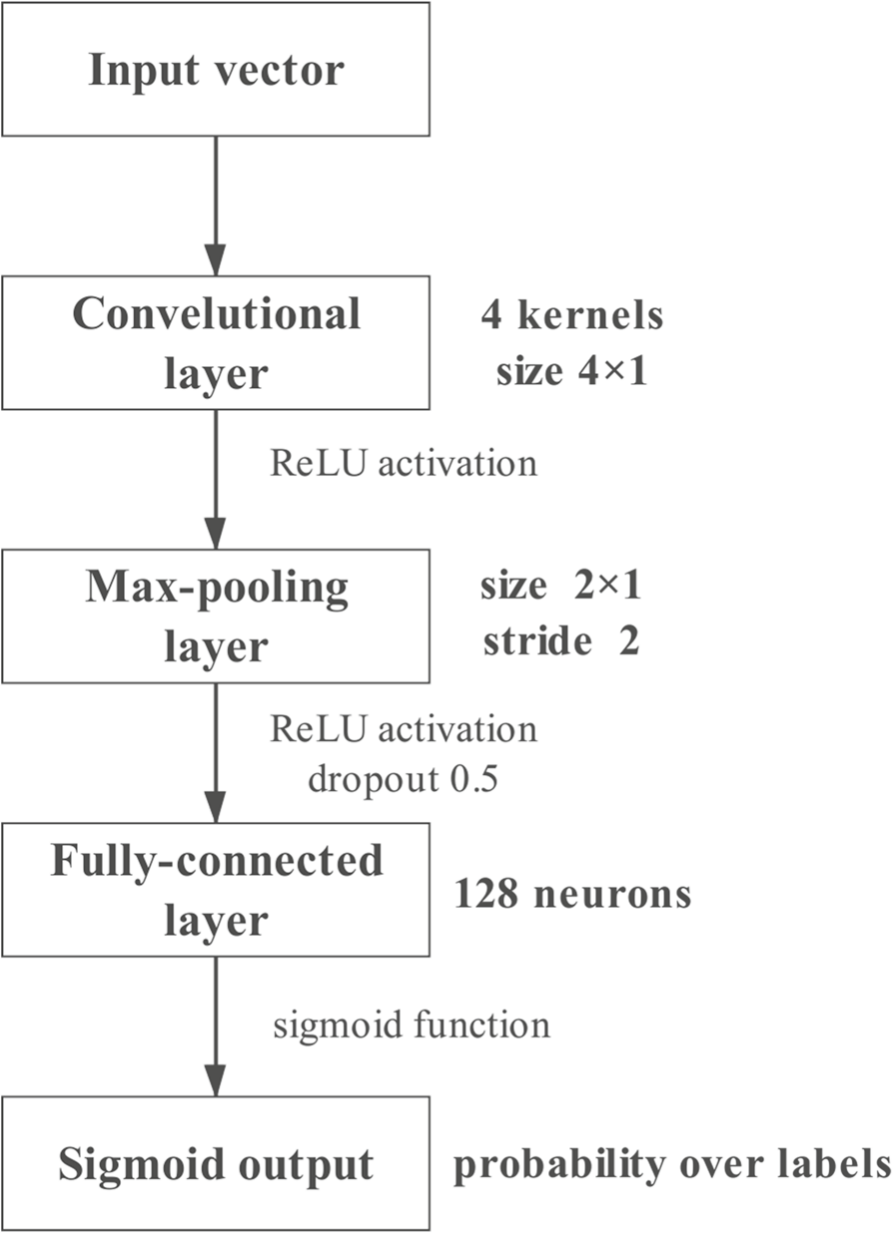
The structure of the convolutional neural network model

Inspired by the success of CNN in classification tasks [34], we use CNN as the supervised learning model and the structure of the prediction model is shown in Fig. 3. The prediction model contains the convolution, max-pooling, fully-connected and output layer. A convolution layer with a rectified linear unit (ReLU) activation fuction [34] is used as a feature extractor [35]. A max-pooling layer is employed to reduce the dimension of features, and the fully-connection layer and the output sigmoid layer are used to classify the tasks.

As the key component of a CNN, convolution layer can help the model to learn local and global structures from the input vector [35]. In our model, the convolutional layer consists of 4 kernels. Given the input vector *X* of length *S*, and the length of weight vector is 4∗1. For each kernel, convolution operation is independent and thus we can obtain four particular feature with the length (*S*−4)+1, which was extracted from the input vector and named feature map *M*. The *M* is obtained as follows:

4 $$ M_{i} = \sum_{j=1}^{4}W_{j}X_{i+j}.  $$

where $(i\in {0,1,\dots,S-4})$, and *W* is initialized by a truncated normal distribution and used as a weight vector. Then a ReLU function is used to the feature map obtained last step:

5 $$ f(x)=max(0,x).  $$

The ReLU function is selected for the excitation function, which can effectively simplify the calculation process and avoid the gradient explosion and disappearance [36].

Next layer is the max-pooling layer, which can extract the maximum value in the pooled region and the pooled region continues to move forward at a certain step size in an input sequence, thus reducing the dimension in each feature map [36]. In our model, the pooled size is 2∗1, and the step size is 2. Given an input vector $A_{i} (i\in {0,1,\dots,S-4})$, the length of output of this layer is $\frac {(S-2)+1}{2}$.

After the first two layers, we use a one-dimensional vector to connect the important features extracted from all the kernels and then pass them to the fully connected layer. The number of hidden units in this layer is 180 and the output of this layer is calculated as follows:

6 $$ f=W*y.  $$

where *W*∈*R*^(*n*∗180)^ is the weight matrix, *y* is the output of pooling layer, and *f* is the ReLU function.

The final output sigmoid layer is constructed for the binary classification. The Sigmoid function maps output values between 0 and 1 for classification, which is extracted by the following equation:

7 $$ S(x)=\frac{1}{1+e^{-x}}.  $$

## Results

### Data preparation

To make the performance evaluationn, DTI-CNN was tested on the drug-target interactions prediction task.

We obtained the heterogeneous network from *Luo*’s paper, which include 12,015 nodes and 1,895,445 edges in total [2]. The isolated nodes are excluded. The heterogeneous network integrates four types of nodes (drugs, proteins, diseases and side-effects) and six type of edges (drug-protein interactions, drug-drug interactions, drug-disease associations, drug-side-effect associations, protein-disease associations and protein-protein interactions) [2]. Based on chemical structures of drugs and primary sequences of proteins, we also built up the multiple similarity networks [16].

The drug nodes, known DTIs and drug-drug interactions were extracted from the DrugBank database [37]. The protein nodes and protein–protein interactions were obtained from the HPRD database [38]. The disease nodes, drug–disease and protein–disease associations were extracted from the Comparative Toxicogenomics Database [39]. The side-effect nodes and drug–side-effect associations were collected from the SIDER database [40].

In our model, we first constructed seven similarity matrices after the Jaccard similarity algorithm. We obtained the drug-related similarity matrices including drug-drug similarity matrix, drug-disease similarity matrix, drug-side-effect similarity matrix and drug similarities matrix. The protein-related similarity matrices include protein-disease similarity matrix, protein-protein similarity matrix and protein similarities matrix. Secondly, we perform RWR algorithm for the two kinds of matrices respectively and splice the single diffusion state matrix of drug and protein networks. After this step, we get two diffusion state matrices corresponding to drug and protein respectively. The rows of the drug diffusion matrix represent different drugs, and the columns represent proteins, diseases, side effects and drugs nodes. The values in the matrix represent the associations between drugs and the four biological entities. The rows of the protein diffusion state matrix represent different proteins. The columns represent proteins, diseases and drugs nodes. The values in the matrix represent the associations between proteins and the three biological entities. Then, we reduce the dimension of drug diffusion state matrix and protein diffusion state matrix respectively by using DAE model. Finally, we obtain the drugs feature vector matrix of 100 dimensions and a total of 708 samples. Similarly, the proteins feature vector matrix is 400 dimensions and 1512 samples.

At last, we adopted the method of ten fold cross validation to divide the train set and test set, in which 90% of the positive and negative samples were used to train model and 10% of the positive and negative samples were used to test the model. According to the known drug-protein interactions matrix, we use the known drug-protein interaction pairs as positive samples. We randomly selected negative samples with the same number of positive samples. In total, we have 3846 samples. After splicing the corresponding protein vectors into drug vectors, we get drug-protein pair vectors of 500 dimensions.

### Model parameters

For the RWR model, the restart probability is 0.5 and the number of iterations is 20.

The parameters used in the DAE model are as follows. For the drug features matrices, the original dimensions are 2832 and the DAE has one hidden layer with 100 units. For the protein features matrices, the original dimensions are 4536 and has one hidden layer with 400 units. For drug and protein features, there are 16 and 32 samples for each batch respectively. The number of epochs is 20. The noise scale value is 0.2 and we use Respro optimizer algorithm to train the model.

The parameters of the CNN model are as follows. We added a dropout layer before the fully-connected layer and the dropout percentage is 0.5. We run 35 epochs with 64 samples for each batch. We used Adam algorithm and set an initial learning rate as 0.001 to optimize the binary cross entropy loss [41].

### Evaluation Metrics

The AUROC and AUPR scores were used to evaluate the model test and comparison. AUROC and AUPR scores are commonly used evaluation criteria for machine learning, which represent the area under the curve of ROC curve and PR curve respectively. The higher the score, the higher the prediction accuracy of the model and the better the performance of the model.

ROC curve refers to the curve with false positive probability (FPR) as horizontal axis and true positive rate (TPR) as longitudinal axis, in which $FPR=\frac {FP}{TN+FP}$, $TPR=\frac {TP}{TP+FN}$. PR curve refers to the curve with Recall as horizontal axis and Precision as longitudinal axis, and $Recall=\frac {TP}{TP+FN}$, $Precision=\frac {TP}{TP+FP}$.

### Performance evaluation on predicting drug-target interactions

**Fig. 4:**
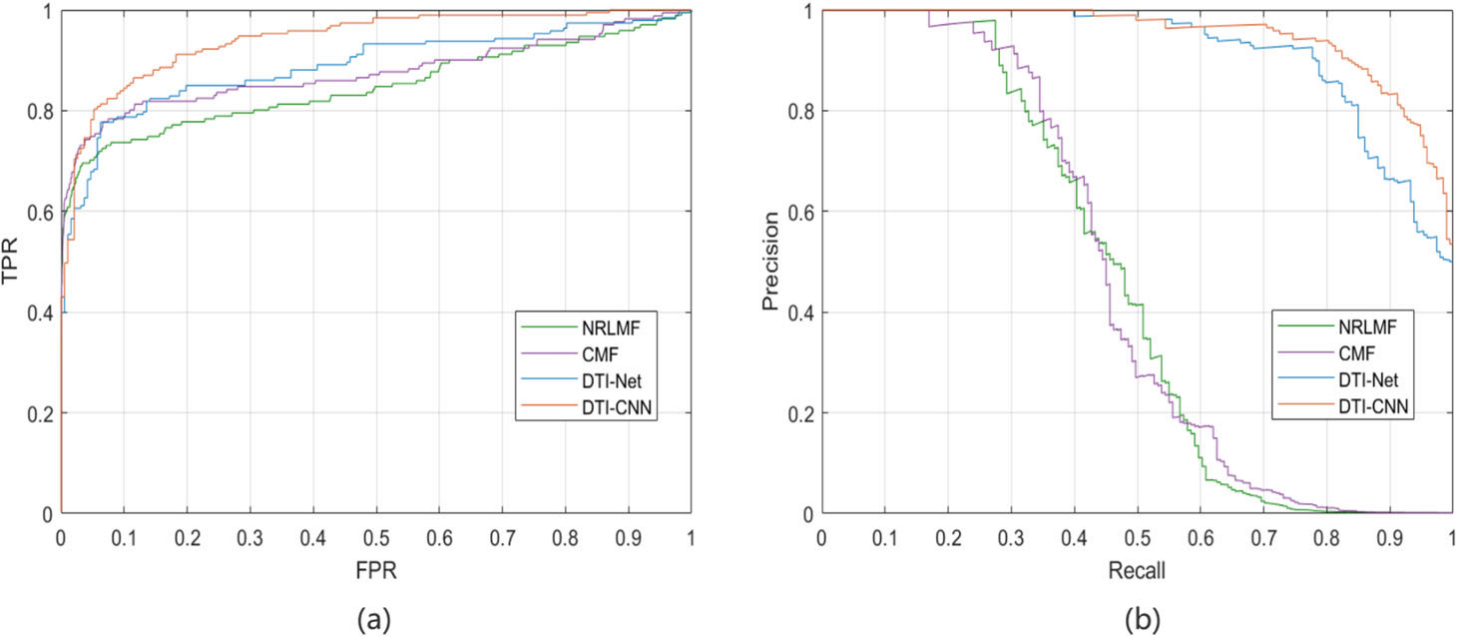
The ROC **(a)** and P-R **(b)** curves of DTI-CNN, DTINet, CMF and NRLMF on drug-target interactions prediction task. The AUROC of DTI-CNN is 0.9416 and the AUPR of DTI-CNN is 0.9499, which performs better on the drug-target interactions prediction task than the other three state-of-the-art DTI prediction methods

We compared the performance of DTI-CNN with three existing state-of-art methods (DTINet [2], CMF [17] and NRLMF [42]) on the task of predicting drug-target interactions.All models are trained and tested with a 10-fold cross validation. Our comparative results are shown in Fig. 4 and Table 1. Comparing with other methods, the result shows that DTI-CNN can perform best on both scores at the same time and DTINet is the second best method. The AUROC of DTI-CNN is 0.9416, which is 0.03 higher than DTINet. The AUPR of DTI-CNN is 0.02 higher than DTINet. In summary, DTI-CNN performs better on drug-target interactions prediction task than the other three state-of-the-art DTI prediction methods.

**Table 1:** The AUROC, AUPR scores of DTI-CNN, DTINet, CMF and NRLMF on drug-target interactions prediction task

Methods	AUROC	AUPR
DTINet	0.9111	0.9290
NRLMF	0.8692	0.4411
CMF	0.9037	0.4173
DTI-CNN	0.9416	0.9499

### Effects of DTI-CNN components

**Fig. 5:**
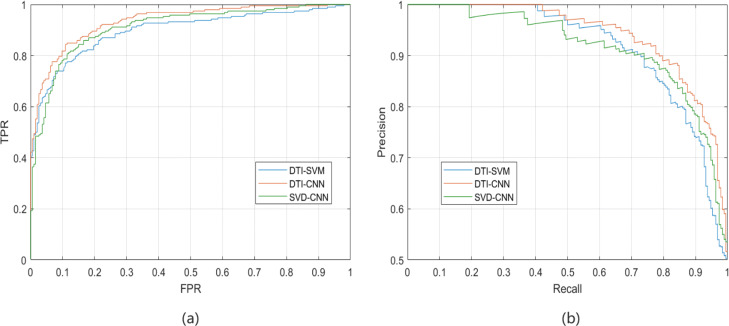
The ROC **(a)** and P-R **(b)** curves of DTI-CNN, SVD-CNN and DTI-SVM on drug-target interactions prediction task. The DTI-CNN achieves both higher AUROC and AUPR scores than the SVD-CNN and the DTI-SVM method, indicating that the DTI-CNN has been appropriately designed

We choose two different approaches in feature selection module and interaction prediction module respectively to figure out the performance of each module of DTI-CNN. To test the effect of our feature selection model, we first use the singular value decomposition (SVD) method [43] to replace our DAE model and name this method as SVD-CNN. To test the effect of the CNN component, we substitute the CNN model with the support vector machine (SVM) model and name this method as DTI-SVM. All three methods are used on DTI prediction task to compare performance and the comparative results are shown in Fig. 5 and Table 2. Comparing with other two methods, the result shows that DTI-CNN can achieve higher AUROC and AUPR scores at the same time, indicating that the two modules of our model are appropriately designed.

**Table 2:** The AUROC, AUPR scores of DTI-CNN, SVD-CNN and DTI-SVM on drug-target interactions prediction task

Methods	AUROC	AUPR
DTI-SVM	0.9068	0.9292
SVD-CNN	0.9306	0.9370
DTI-CNN	0.9416	0.9499

### Case study in three drugs

We extracted the known DTIs from the DrugBank database. We choose the three drugs with the largest number of interactions in known DTIs, which are Quetiapine, Olanzapine and Meprobamate. In the train set, we exclude all the features and interactions between the three drugs and their related proteins to avoid logic circle. In the test set, we input the features of the three drugs and its related proteins. In the “Quetiapine set”, 24 of 24 known interactions are identified. In the “Meprobamate set”, 23 of 24 known interactions are discovered. And in the “Olanzapine set”, 23 of 23 known interactions are recognized. These results indicate that the DTI-CNN method has a good performance on drug-target interactions prediction.

## Discussion

At present, there are three traditional kinds of DTI prediction approaches which contains molecular-based approach, ligand-based approach and network-based approach [10]. There are a variety of related models, and the demand for algorithm prediction accuracy is getting higher and higher. The feature learning approach based on deep learning is different from the traditional neural network [44]. Through layer-by-layer learning, we can learn the essential features of the data set without relying on a large scale of samples., so as to predict the unknown data more accurately [45].

The purpose of this research is to improve the prediction accuracy by using the CNN model based on depth learning on the basis of the developed method DTINet. The DTINet contains two modules which are feature extraction and classification. In the first module, we replace the dimension reduction model based on SVD with DAE model to ensure that we can learn features that are more suitable for neural network learning. Then, we improve the classification prediction model of DTINet and use the CNN model as the new prediction model in the second module. Compared with the IMC approach used by DTINet, the CNN model can take into account the topological information and interrelation between the nodes in the network. In addition, we also choose three state-of-the-art DTI prediction methods as comparison. The experimental results show that the AUROC and AUPR scores of our model are both higher than DTINet, NRLMF and CMF.

In addition, we want to know which network contributes more to the DTI prediction. We sequentially remove a network from the original heterogeneous networks as new input data, and then use our DTI-CNN method to perform DTI prediction. The results are shown in Table 3. The result shows that the drug-drug interaction of drug networks and the protein similarities of protein networks contributed more to the DTI prediction. When the drug-drug interaction network was removed, the result achieves both the lowest AUROC and AUPR scores at the same time.

**Table 3:** The AUROC, AUPR scores of sequentially strip out a network from the original heterogeneous network as new input data of DTI-CNN on drug-target interactions prediction task

Networks	AUROC	AUPR
Without drug-drug	0.9299	0.9370
Without drug-disease	0.9343	0.9416
Without drug side-effect	0.9344	0.9444
Without drug similarities	0.9345	0.9425
Without protein-protein	0.9418	0.9499
Without protein-disease	0.9364	0.9452
Without protein similarities	0.9327	0.9411

In the future, we will consider adding more relevant information to the heterogeneous network. For the CNN model, we can add the network structure appropriately to accommodate more complex input networks. In this work, although DTI-CNN is mainly designed to predict DTIs, it is an extendible method and can also be used to predict other related directions in the future, such as drug-drug, drug-side-effects and protein-disease.

## Conclusion

In this paper, we propose a learning-based method named DTI-CNN to predict the drug-target interactions. Firstly, the Jaccard similarity coefficient and RWR model are used to obtain the relevant features of drugs and targets from heterogeneous networks. Then, we use DAE model to reduce dimensions and identify the essential features. Thirdly, based on the features obtained from the last step, a CNN model is constructed to make a prediction of DTIs.To demonstrate the advantages of DTI-CNN, we compare it with three advanced methods. In addition, we also evaluate the effect of each DTI-CNN module. All the experimental results show that the performance of DTI-CNN is better than that of the existing methods and the proposed method is appropriately designed. The case study also shows that DTI-CNN can be used to predict the drug-target interactions.

## Abbreviations

DTIs: Drug-target interactions
IMC: Inductive matrix completion
CMF: Cooperation matrix factorization
CNN: Convolution neural networks
RWR: Random walk with restart
DAE: Denoising autoencoder
MSE: Mean-square error
BP: Backpropagation
ReLU: Rectified linear unit
FPR: False positive probability
TPR: True positive rate
SVD: Singular value decomposition
SVM: Support vector machine.
